# Inflammatory Mechanisms of Idiopathic Epiretinal Membrane Formation

**DOI:** 10.1155/2013/192582

**Published:** 2013-11-11

**Authors:** Malav Joshi, Shivi Agrawal, John Byron Christoforidis

**Affiliations:** Department of Ophthalmology, University of Arizona Medical Center, Tucson, AZ 85711, USA

## Abstract

The pathogenesis of idiopathic epiretinal membranes (iERMs), a common pathology found in retina clinics, still eludes researchers to date. Ultrastructural studies of iERMs in the past have failed to identify the cells of origin due to the striking morphologic changes of cells involved via transdifferentiation. Thus, immunohistochemical techniques that stain for the cytostructural components of cells have confirmed the importance of glial cells and hyalocytes in iERM formation. The cellular constituents of iERMs are thought to consist of glial cells, fibroblasts, hyalocytes, etc. that, in concert with cytokines and growth factors present in the vitreous, lead to iERM formation. Recently, research has focused on the role of the posterior hyaloid in iERM formation and contraction, particularly the process of anomalous PVD as it relates to iERM formation. Recent advances in proteomics techniques have also elucidated the growth factors and cytokines involved in iERM formation, most notably nerve growth factor, glial cell line-derived growth factor, and transforming growth factor **β**1.

## 1. Introduction

Epiretinal membranes (ERMs) are classified as idiopathic when they are not associated with any other ocular disease processes such as retinal detachment, intraocular inflammation, trauma, and retinal vascular diseases. Idiopathic epiretinal membranes (iERMs), whose exact pathogenesis still remains unknown, are characterized by the growth of fibrocellular tissue on the inner limiting membrane (ILM). They can range from subtle cellophane-like films without visual consequences to markedly contractile membranes that can cause metamorphopsia and decreased visual acuity [1]. Several theories of the responsible pathologenic mechanisms have been proposed, including the role of glial cells, fibroblasts, hyalocytes, and so forth assisted by cytokines and growth factors present in the vitreous fluid; however, the debate regarding the types of cells that produce iERMs and the means by which they reach the retinal surface has continued for decades. With recent improvements in imaging techniques coupled with immunocytochemistry and proteomic techniques, the understanding of the development of iERMs has evolved. This review summarizes the prior and latest developments in understanding the inflammatory mechanisms of idiopathic ERMs.

## 2. Cellular Constituents in iERM Formation

During the past decades, significant progress has been made elucidating the pathogenic mechanisms in iERM formation; however, many fundamental questions still remain unanswered. One of the significant impediments to a greater understanding of how and why iERMs occur is the accurate identification of the cells that participate. Morphologic analysis of surgically excised ILM specimens has demonstrated a variety of cells in iERM, including glial cells (Müller cells, fibrous astrocytes, and microglia), hyalocytes, retinal pigment epithelial (RPE) cells, fibroblasts, and myofibroblasts [2]. However, because the cells in the vitreous commonly undergo striking morphologic changes via transdifferentiation, morphologic criteria alone have proven inadequate for identifying the origin of cells [3]. In fact, Vinores et al. confirmed that when glial cells, fibroblasts, and RPE cells are cultured on vitreous, they undergo time-dependent changes in morphology and are essentially indistinguishable from each other by ultrastructural criteria [4]. Therefore, recent research has focused on using immunohistochemical markers of structural proteins such as intermediate filament proteins to assist in cell-type determination.

Commonly used antibodies against structural proteins and their respective target cells are listed in Table 1. Glial cells usually predominate in iERMs with little traction while myofibroblasts are the major cell type in membranes with significant traction [4]. In a study by Zhao et al., Müller cells and hyalocytes were found to be the predominant cell type in macular pucker specimens. All surgically removed iERM specimens were found to have positive immunostaining for glial fibrillary acidic protein (GFAP), CD45, CD68, CD163, vimentin, and cellular retinaldehyde binding protein (CRALBP), indicating the presence of glial cells and hyalocytes [5]. Kir4.1 was also found in iERMs, which is reported to be found on Müller cell end-feet membranes [6]. Immunostaining for pan-cytokeratin was negative, predicting little if any role of RPE cells in iERMs. The importance of Müller cells is highlighted by the fact that all immunomarkers for Müller cells were positive in this study including GFAP, CRALBP, vimentin, and Kir4.1. All hyalocyte markers were also immunopositive. Interestingly, this study also found colocalizations of GFAP and hyalocyte markers CD45 and CD163 in 20% of specimens. These double-labeled cells may represent hyalocytes since hyalocytes with positive GFAP expression have already been described in other species [7, 8]. Hyalocytes are considered to be of macrophage lineage, so they could have phagocytosed GFAP positive debris or apoptotic cells, which could explain their immunopositivity for GFAP [9]. Colocalization of CD163 and *α*-SMA was also seen in single cases, which most likely indicated hyalocytes that might have transdifferentiated into myofibroblast-like cells. These results support the hypothesis that hyalocytes and Müller cells constitute the major cell type in iERM.

A study by Schumann et al. also confirmed the presence and importance of Müller cells as a component of iERM [2]. They tested surgically excised flat-mounted ERM specimens for GFAP, hyalocyte markers (CD45 and CD64), vimentin, CRALBP, and *α*-SMA. They also found the colocalization of GFAP and the hyalocyte markers, which are presumed to be hyalocytes that could have phagocytosed GFAP positive debris or apoptotic cells. Additionally, cells colocalized with GFAP/vimentin and GFAP/CRALBP were also found and thought to represent Müller cells, and, finally, cells positive for GFAP but not for hyalocyte markers conceivably signified Müller cells as well. Thus, these findings also highlight the importance of Müller cells in ERM proliferation.

The importance of glial cells in iERM formation cannot be denied. However, there is disagreement when it comes to deciding which type of glial cell, Müller cells versus astroglia, is the major cell type involved. According to Foos, it is unlikely that ERMs derive from Müller cells since they are anchored in the outer retina and attached to photoreceptor cells [10]. On the other hand, Kase et al. claim that Müller cells and their processes are the main constituent cells in iERMs [11]. They used immunohistochemical staining for glutamine synthetase (GS) (expressed specifically in Müller cells and processes and not astrocytes) on surgically excised iERMs. All the iERMs demonstrated a continuous, isodense pattern of immunoreactivity for GS, indicating that Müller cells are the main cell type responsible for iERM formation, not astrocytes. However, due to the continuous appearance of GS immunoreactivity in the collagenous tissues of the ERMs, these most likely represented extensions of Müller cell processes through the ILM, not the actual Müller cells as a whole. There was also a minor part of the ERM that showed no immunoreactivity for GS, which likely represented hyalocytes, myofibrocytes, and so forth. Rentsch also believes that Müller cell processes, not the entire cells, extend into the vitreous cavity through the ILM and serve as scaffolds for the migration and proliferation of other cells [12].

Hyalocytes, named for their location in the posterior hyaloid, are considered to be one of the macrophage lineages, and accumulating evidence has emphasized the importance of their role in iERM formation. In a study by Kohno et al. [9], immunohistochemistry performed on surgically excised iERMs demonstrated the presence of GFAP and *α*-smooth muscle actin (*α*-SMA) immunopositive cells in all ERMs. GFAP is an intermediate filament protein that is found in glial cells, while *α*-SMA is thought to be an intermediate filament protein presumed to be essential for extracellular matrix contraction by fibroblasts [13]. Interestingly in the study, the *α*-SMA positive cells were located mainly at the contracted focus of the ERM, while the GFAP positive cells were present at the peripheral, noncontracted areas of the ERM in all samples. In order to figure out whether these *α*-SMA positive cells were transdifferentiated hyalocytes or glial cells, a collagen gel contraction assay was performed using cultured bovine hyalocytes or normal human astrocytes to evaluate the contractile property of the cells in the presence of transforming growth factor *β*2 (TGF *β*2). TGF *β*2 is thought to stimulate transdifferentiation of cells into myofibroblasts. The bovine hyalocytes showed strong contractile activity of collagen gels and overexpression of *α*-SMA in the presence of TGF *β*2 while the human astrocytes did not. One can deduce from this that hyalocytes, which transdifferentiate into myofibroblasts in the presence of TGF *β*2, might play an important role in ERM contraction. The authors also claimed that the GFAP positive cells found in the periphery were astrocytes not Müller cells. When they stained for a Müller cell marker (antiglutamine synthetase), they only occasionally detected immunopositive cell processes and no obvious Müller cell aggregation was seen. Thus, one can surmise that while Müller cell processes might play a role in ERM formation, the majority of GFAP positivity seen in iERMs represents accessory glia [9].

The potential for transdifferentiation to myofibroblasts is not exclusive to hyalocytes. In a study done by Guidry [14], who evaluated Müller cells as potential sources of contractive cells in proliferative diabetic retinopathy, Müller cells were shown to lose their GFAP and GS expression with concurrent increase in *α*-SMA immunoreactivity, thereby demonstrating transdifferentiation into myofibroblast-like cells capable of contractile properties.

An interesting finding in a study by Lesnik Oberstein et al. suggested the possible role of retinal ganglion cells in iERM formation. Their study demonstrated neurofilament processes of ganglion cells in all of the 32 iERMs examined by immunohistochemistry. The ERMs were labeled with antibodies for neurofilament protein, presumed to originate from retinal ganglion cells. Previous studies on feline retina have shown that neurites from ganglion cells and horizontal cells can be found after experimental retinal detachment next to Müller cells [15]. However, in these cases, the growth of these neurites was thought to result from a reaction to retinal injury. This study showed growth of neurites into iERMs with no history of trauma in the patients. Interestingly, these neurites were only found in regions where Müller cells were present, suggesting that some type of signal from Müller cells stimulates the ganglion cells to sprout neurites. However, the reason for this type of neurite growth is not known. In fact, in a series by Parolini et al., in which ERMs were removed from patients with idiopathic lamellar holes, antineurofilament staining was not demonstrated [13].

## 3. Pathophysiology of Idiopathic ERMs

Structurally speaking, there are two types of iERMs that have different clinical presentations: simple and tractional ERMs. Simple ERMs are membranes with delicate cellophane-like films on the internal limiting membrane (ILM) with mild to no visual symptoms. These membranes are usually composed mostly of glial cells. On the other hand, tractional iERMs are thicker with contractile properties that cause surface wrinkling of the retina and are usually accompanied by decreased vision and metamorphopsia. They are composed of glial cells plus contractile cells [16]. The two main components of an ERM are extracellular matrix structures such as fibronectin and collagen and cells of extraretinal and retinal origin such as glial cells, fibroblasts, and hyalocytes [17].

The complete pathogenesis of iERM is unknown, but many theories have been proposed. The most widely accepted theory is that iERM is a consequence of surface breaks formed in the ILM by posterior vitreous detachment (PVD) that allows glial and other cells from the underlying retina to migrate through the defect and proliferate on the ILM [1]. Some of the original studies of iERMs were performed by Foos, in which he carried out an ultrastructural study of 8 cases of simple ERMs using an electron microscope. In his studies, he found simple ERMs to only contain glial cells. He also hypothesized that an initial event damages the superficial retina and leads to glial cell proliferation and migration through the defect. Because of the defect on the retinal surface, glial cells react through extension and hypertrophy of their processes in an effort to repair the defect. Meanwhile, some of the other cells divide and contribute to the substance of the ERM. He goes on to say that the breaks in the ILM, following the formation of ERM, can heal and make it difficult to find them later on [10].

In more recent years, attention has been placed on attempting to understand the role of vitreous in iERM formation, since most cases of iERM seem to occur in patients with a PVD. In fact, Foos [18] reported the presence of condensed collagen fibrils indistinguishable from vitreous collagen in premacular fibrosis. Bellhorn and colleagues [19] also identified vitreous in variable amounts within the ERM of a lesion they studied with electron microscopy. The role of vitreous was further elucidated when Kishi and Shimizu [20] reported oval or round defects in detached posterior hyaloid membranes of patients with idiopathic preretinal fibrosis. They postulated that a premacular oval defect in the detached posterior hyaloid membrane plays a key role in the development of idiopathic preretinal macular fibrosis. In their study, they found that 31 (65%) of 48 eyes with a PVD and idiopathic preretinal macular fibrosis had an oval or round defect and 12 (25%) of 48 eyes had a break in the premacular area. This implies that a majority of the eyes with a PVD and a defect in the premacular cortical vitreous develop idiopathic preretinal macular fibrosis. The theory is that in some cases the posterior cortical vitreous may remain attached to the retina during PVD development, which leads to the defect in the premacular detached cortical vitreous. But more importantly, it is the remnants of the cortical vitreous on the premacular ILM that then serve as a structural component and provide a medium upon which glial cells and hyalocytes can proliferate to form an iERM. Histologic studies have supported this theory and have shown that a portion of posterior cortical vitreous does remain attached to the premacular ILM after a PVD [21]. Hikichi et al. [22] conducted an in vivo study to further elucidate the relationship between premacular cortical vitreous defects and their relationship to idiopathic premacular fibrosis. They also found that the incidence of the defect in the detached premacular cortical vitreous was significantly higher in eyes with idiopathic premacular fibrosis than in eyes without. However, 27 (75%) of 36 eyes with premacular fibrosis did not exhibit the defect in the premacular cortical vitreous.

It was Sebag who later unified this concept and coined it anomalous PVD. According to Sebag, for an uncomplicated PVD to occur, two processes must occur concurrently: weakening of vitreoretinal adhesion and vitreous liquefaction [23]. An anomalous PVD occurs when the extent of vitreous liquefaction exceeds the degree of weakening of vitreoretinal adhesion and leads to posterior vitreoschisis. This is when splitting of the posterior cortical vitreous occurs and forward displacement of the vitreous body leaves the outer layers of posterior vitreous cortex (which contains hyalocytes) still attached to the retina, potentially resulting in the formation of macular pucker. Exactly how these hyalocytes cause ERM is not known, but, according to Kampik, these hyalocytes stimulate Müller cells to send processes through an intact ILM to form the scaffolding which allows other cells to then be taken up into the membrane [24]. Sebag has proposed pharmacologic vitreolysis as a way to weaken the vitreoretinal adhesion to safely detach the posterior vitreous cortex and prevent an anomalous PVD [23].

In a order to validate the theory of anomalous PVD as the initiating event for the formation of iERM, Sebag et al. studied 44 eyes with macular pucker using combined optical coherence tomography and scanning laser ophthalmoscopy to look for vitreoschisis. Vitreoschisis was detected in 19 out of 44 eyes (43.2%) with macular pucker. The authors considered vitreoschisis to be present only when they saw two membranous layers of the posterior vitreous cortex join into one, forming a “Y” shaped configuration. However, the authors stated that there were many cases where a clear cut “Y” shape was not seen but a distinct thin membrane of posterior vitreous cortex was visible anterior to the surface of the retina [25]. Future studies with high-resolution OCTs are needed to investigate whether the incidence of vitreoschisis is even greater than that observed in this study.

Going along with this theory, Kampik also believes that the role of vitreoschisis is likely responsible for iERM formation. In the many specimens he has examined, he has rarely encountered a break in the ILM, and it is therefore unlikely to be a mechanism for iERM formation. According to his findings, there are two types of iERM membranes: type I is when there is vitreous collagen sandwiched between the ILM and the ERM, and type II is when the cells proliferate directly on the ILM surface with sparse or no collagen layer in between [24]. Since the posterior vitreous cortex is composed of many thin lamellae, very few delicate lamellae would actually be present on the ILM if the vitreoschisis occurs in the very posterior portion of the cortex. This would explain why, in type II iERMs, there is sparse to no vitreous seen in some areas.

One can speculate that pharmacologic vitreolysis could potentially have a therapeutic role in type I iERMs, whereby the enzyme plasmin could act and detach the ERM. However, this would not be possible in type II membranes. Surgically speaking, this could explain why some membranes are easier to peel than others. Additionally, one would have to peel two membranes to prevent recurrence and to rid sources causing traction in type I membranes, whereas, in type II membranes, one would only have to peel one membrane—the ILM. In fact, in a study by Gandorfer et al., simple ERM removal leaves 20% of total cell count behind on the ILM in 2 of 3 patients with iERM [26]. These most likely represent type I membranes and peeling just the ERM in these patients would leave the residual cells to proliferate and cause ERM recurrence. Thus, ILM staining with subsequent removal is important for recurrence prevention. According to Kampik, the cells need the scaffolding of either the ILM or the native vitreous in order to proliferate, and by peeling the ILM both are taken away [24]. Kenawy et al. reported the difficulty of removing the ILM in the presence of ERM due to the deeper cleavage plane in ILM peeling [27]. Interestingly, they found that patients with ERMs tended to have glial and/or neuronal cells on the retinal surface as well as the vitreous surface of the ILM. These cells found on the retinal surface of the ILM account for the deeper cleavage plane in ILM peeling. This study also suggests that iERM formation, which previously was considered to be predominantly epiretinal, may have a significant intraretinal component.

## 4. Cytokines and Growth Factors

There is very limited data available on the proteomics of iERMs alone. However, according to a study by Mandal et al. [1], high abundance proteins found in undiluted vitreous samples from patients with iERMs include *α*-antitrypsin, apolipoprotein A-1, transthyretin, and serum albumin. They also compared these results to vitreous samples from patients with idiopathic macular holes and found no significant difference between the two. This leads one to speculate that both iERM and macular holes involve similar inflammatory processes.

Since it is known that glial cells are one of the most important cellular components of iERM, understanding the role of molecules involved in glial signal transduction is important. Basic fibroblast growth factor (bFGF) is such a molecule. It supports the survival and maturation of both neurons and glial cells and may play an important role in the regeneration after neural injury [28]. In a study by Harada et al. [29], polymerase chain reaction (PCR) analysis revealed bFGF mRNA expression in 10 of 15 (67%) iERMs and 13 of 19 ERMs from proliferative diabetic retinopathy (PDR) patients. Chen et al. [30] found bFGF immunoreactivity in five of seven (71%) iERMs and four of eight (50%) PDR membranes.

Nerve growth factor (NGF) and glial cell line-derived neurotrophic factor (GDNF) also may be involved in ERM formation. In the same study by Harada et al. [29], they examined the expression of receptors for neurotrophins (trkA, trkB, trkC, and p75^NTR^) and GDNF (GFR*α*1, GFR*α*2, and Ret) in ERMs obtained from PDR and iERM patients. Expressions of neurotrophin receptor mRNAs were similar in both groups. A study by Iannetti et al. [31] also studied the role of NGF in iERMs and found the levels to be significantly higher in iERM samples versus control groups (patients without ERMs who underwent vitrectomy for primary retinal detachment within 72 hours of onset). In terms of GDNF, the expression of GFR*α*1 receptor mRNA was surprisingly higher in iERMs (12 of 15 cases) compared to PDR ERMs (eight of 19 cases). On the other hand, GFR*α*2 expression levels were significantly higher in PDR ERMs (17 of 19 cases) versus iERM (two of 15 cases). Despite the above findings, few other studies have shown GDNF levels to be far below the sensitivity threshold in iERM samples [31, 32]. The discrepancy in the study results might relate in part to the methods used to process the samples (i.e., ELISA versus PCR). Further studies are necessary in order to elucidate the role GDNF and neurotrophins play in iERM formation.

Recently, a more advanced technique for proteomics using liquid chromatography mass spectrometry and multiplex protein assays was utilized by Pollreisz et al. [17] to study the aqueous and vitreous fluids from patients with iERMs. The majority of proteins identified were involved in the classical and alternative pathway of complement activation, proteolysis, and cell adhesion. Most of the proteins were found in similar quantity between the aqueous humor and vitreous sample; however, there were 8 proteins that were expressed at a lower level in the aqueous fluid compared to vitreous fluid. Of these proteins, fibrinogen A was the most highly expressed protein in the vitreous compared to the aqueous fluid. Fibrinogen has been implicated in the development of vitreous membrane formation in a rat model [33]. The other 7 proteins have not been reported to play a role in iERM formation. Multiplex protein array analysis showed similar concentrations of cytokines and growth factors in the aqueous versus vitreous fluids, except for platelet-derived growth factor A (PDGF-A). This factor was expressed at a higher level in the vitreous fluid. Cassidy et al. have reported higher levels of PDGF in vitreous fluids of eyes with proliferative vitreoretinopathy after retinal detachment compared to healthy controls [34].

Nerve growth factor (NGF) and transforming growth factor *β*1 (TGF*β*1) both play a crucial role in fibroblast activities. According to a study by Minchiotti et al., both TGF *β*1 and NGF mRNA were found in all 8 iERMs evaluated [35]. In fact, every iERM displayed *α*-smooth muscle actin (*α*-SMA) positive myofibroblasts that expressed NGF and its receptors *trkA* ^NGFR^ and p75^NTR^. Biologic effects of NGF include fibroblast migration, differentiation into myofibroblasts, and extracellular matrix contraction. Thus, it is reasonable to suppose that TGF *β*1 and NGF could target glial cells and stimulate them to transdifferentiate into myofibroblasts and could also stimulate myofibroblasts to turn on their contractile actions.

The study by Iannetti et al. also reported the role of transforming growth factor *β*1 (TGF*β*1), *β*2, and nerve growth factor (NGF) in the pathogenesis of iERM [31]. They reported much higher TGF*β*2 levels in patients with iERMs compared to controls, whereas the levels of TGF*β*1 was similar to controls. This is in contradiction with what was reported by Minchiotti et al., which reported TGF*β*1 expression in all iERM specimens. According to Iannetti, TGF*β*2 is the most important growth factor in the pathogenesis of iERM and possibly stimulates the differentiation of specific types of glial cells or hyalocytes into myofibroblasts, inducing ERM contraction. The previously reported study by Kohno et al. also demonstrated the importance of TGF*β*2 in iERM contraction [9]. One can speculate on the efficacy of therapeutic agents against TGF*β*2 in preventing iERM formation and contraction.

Vascular endothelial growth factor (VEGF), one of the most extensively studied vitreoretinal growth factors, has also been reported in iERMs. In a study by Mandelcorn et al., 11 (85%) of 13 iERMs stained positively for VEGF, but there was no statistically significant relationship between the presence of VEGF and leakage on fluorescein angiogram [36]. Positive VEGF immunoreactivity of iERMs was also found in a study by Chen et al. [30]. However, since retinal glia have been known to produce VEGF, this is not surprising [37]. What is puzzling is why there are no blood vessels in iERM despite the presence of VEGF. One possibility is that there are other cells in the iERM besides endothelial cells that are targeted by VEGF. It is also plausible that the presence of endothelial growth inhibitory factors, such as TGF-*β*, may prevent VEGF from exerting its angiogenic activity [38].

## 5. Conclusion

Despite the advances in imaging technology, immunohistochemistry, and proteomics, the exact mechanism of iERM formation is still unclear. We have come a long way in understanding the cell types involved, but much of our understanding related to the interdependence of cytokines and growth factors involved in iERM production is incomplete. Further studies are needed to evaluate the cells, cytokines, and growth factors involved in iERM formation.

## Figures and Tables

**Table 1:** Antibodies used for immunocytochemical staining.

Antibodies	Target cells/structure
Glial fibrillary acidic protein (GFAP)	Glial cells
Vimentin	Glial cells
Cellular retinaldehyde binding protein (CRALBP)	Glial cells/Retinal pigment epithelial cells
Kir4.1	Muller cell end-feet membranes
CD 45	Hyalocytes
CD 64	Hyalocytes
CD 168	Hyalocytes
Pan-cytokeratin	Retinal pigment epithelial cells
Neurofilament	Retinal ganglion cells
*α*-Smooth muscle actin (*α*-SMA)	Fibroblasts, Myofibroblasts
CD 68	Macrophages and Microglia
